# Mutations close to a hub residue affect the distant active site of a GH1 β-glucosidase

**DOI:** 10.1371/journal.pone.0198696

**Published:** 2018-06-06

**Authors:** Valquiria P. Souza, Cecília M. Ikegami, Guilherme M. Arantes, Sandro R. Marana

**Affiliations:** Departamento de Bioquímica, Instituto de Química, Universidade de São Paulo, São Paulo, SP, Brazil; Griffith University, AUSTRALIA

## Abstract

The tertiary structure of proteins has been represented as a network, in which residues are nodes and their contacts are edges. Protein structure networks contain residues, called hubs or central, which are essential to form short connection pathways between any pair of nodes. Hence hub residues may effectively spread structural perturbations through the protein. To test whether modifications nearby to hub residues could affect the enzyme active site, mutations were introduced in the β-glycosidase Sfβgly (PDB-ID: 5CG0) directed to residues that form an α-helix (260–265) and a β-strand (335–337) close to one of its main hub residues, F251, which is approximately 14 Å from the Sfβgly active site. Replacement of residues A263 and A264, which side-chains project from the α-helix towards F251, decreased the rate of substrate hydrolysis. Mutation A263F was shown to weaken noncovalent interactions involved in transition state stabilization within the Sfβgly active site. Mutations placed on the opposite side of the same α-helix did not show these effects. Consistently, replacement of V336, which side-chain protrudes from a β-strand face towards F251, inactivated Sfβgly. Next to V336, mutation S337F also caused a decrease in noncovalent interactions involved in transition state stabilization. Therefore, we suggest that mutations A263F, A264F, V336F and S337F may directly perturb the position of the hub F251, which could propagate these perturbations into the Sfβgly active site through short connection pathways along the protein network.

## Introduction

The Protein Structure Network (PSN) is an interesting tool for analysis of the tertiary structure of proteins [1]. The approach permits a global analysis of the complete set of noncovalent interactions in the protein structure, resulting in a compact view of the role of each residue within the structure network. PSN representations improve the analysis of specific residues and their interactions, avoiding biased choices among the myriad of contact sets that can be built from the protein structure [1].

PSNs have “small-world” properties, exhibiting high local connectivity, a high clustering coefficient (C) and a small average shortest contact pathway (L, also known as path length). A small number of contacts (usually around 3 to 5) is enough to link any two protein residues, even those distant through structure [2–7].

Two centrality parameters calculated from the PSN are useful to characterize the relative importance of individual residues in establishing short contact pathways: betweenness centrality is the frequency a given residue is among the shortest contact pathways through the protein structure; closeness centrality presents the average number of contacts one residue performs with the remaining protein. Residue centrality can also be evaluated by the increase in path length when a specific residue is removed from the PSN [6].

The PSN approach has revealed that core residues in enzyme active sites [8] and allosteric pathways [9–14] have high closeness centrality. Point mutations directed to the same contact pathway have an additive effect [15; 16], whereas mutations directed to residues connecting to the same active site region, exhibit coherent qualitative effects on enzyme activity [17]. These observations suggest that central residues of the PSN may indicate regions or residues which spread perturbations more easily along the protein structure. Thus, measures of residue centrality could also be combined with structural analysis to help identifying residues involved with the protein function.

Sfβgly is a GH1 β-glucosidase from the fall armyworm *Spodoptera frugiperda* that has a (β/α)8 barrel fold and an active site composed of 4 subsites (-1, +1, +2 and +3) located in a pocket at the top of the central barrel (PDB ID: 5CG0) [18; 17]. Sfβgly catalyzes the hydrolysis of *p*-nitrophenyl β-glycosides and oligocellodextrins [18] using a double-displacement mechanism in which E187 is the catalytic acid and E399 is the catalytic nucleophile [19; 20]. Subsite +1, closer to the active site entrance, is formed by residues W371, E190, E194 and K201. The contribution of the latter three residues to the specificity of binding the substrate aglycone has been previously characterized [21]. Subsite -1, located in the bottom of the active site pocket, interacts with the substrate glycone, which may be glucose, galactose or fucose. Residues Q39 and E451 are essential to the subsite -1 specificity for the substrate glycone due to hydrogen bonds with the hydroxyl groups 4 and 6 of the monosaccharidic glycone [22–24].

The PSN of Sfβgly has “small-world” properties and presents 11 hub residues (R97, F251, S358, E399, T245, K366, F334, S247, N249, Y420 and Y331) identified by their relevance to the shortest path length [25] (S1 Table). Removal of these residues from the PSN resulted in path length increments significantly higher than the average increament (S1 Table) [25]. Among these hub residues, F251 is the highest ranked among the eight hubs (F251, S358, T245, K366, F334, S247, N249 and Y420) placed outside of the active site (S1 Table). Removal of F251 from the PSN increases the path length in 6 standard deviations. Consistently, experiments have shown that point mutantions directed towards hub residues as well as towards residues surrounding F251 decreased thermal stability of Sfβgly [25].

Here we analyze the effect on Sfβgly enzymatic activity of mutations in the region surrounding F251, which is approximately 14 Å distant from the active site (the catalytic nucleophile E399 was taken as reference point for the active site). Effects of mutations were probed by determining steady-state kinetic parameters for substrate hydrolysis (*p*-nitrophenyl β-glycosides and cellobiose) and, based on them, the stability of the enzyme-transition state (ES^‡^) complex. Therefore, we evaluated how effectively mutations around a PSN hub affect a distant active site.

## Materials and methods

### Expression and purification of the wild-type and mutant Sfβgly

The pET46 vectors (Merck Millipore; Billerica, MA, USA) encoding for the wild-type and mutant Sfβgly were previously described [25]. Recombinant enzymes were produced in NovaBlue (DE3) bacteria using the previously described protocol [25]. The enzyme purification was performed using nickel-nitrilotriacetic acid resin (Qiagen, Valencia, CA, USA) [25]. Samples of the purified enzymes were submitted to buffer exchange using HiTrap desalting columns (GE HealthCare, Little Chalfont, UK) following the manufacturer instructions. Circular dichroism and tryptophan fluorescence spectra were employed to check the proper folding of the purified enzymes [25]. The protein concentrations were determined from the absorbance at 280 nm in the presence of 6 M guanidium hydrochloride prepared in 50 mM sodium phosphate at pH 6.5. The extinction coefficients of the wild-type and mutant proteins were calculated as previously described [26; 27].

### Enzyme kinetic analysis

The initial rates for the hydrolysis of the *p*-nitrophenyl β-glycosides (NPβglc, *p*-nitrophenyl β-glucoside; NPβgal, *p*-nitrophenyl β-galactoside; NPβfuc, *p*-nitrophenyl β-fucoside) and cellobiose were determined at 30°C using substrates prepared in 50 mM sodium citrate–sodium phosphate buffer at pH 6. The hydrolysis of the *p*-nitrophenyl β-glycoside substrates was detected by following the production of *p*-nitrophenolate, whereas hydrolysis of cellobiose was detected by the glucose production. The production of two glucoses from one cellobiose was taken into account in the calculations. At least ten substrate concentrations, bracketing the *K*_m_ values, were used for the determination of the enzyme kinetic parameters (*K*_m_ and *k*_cat_). Rate and substrate concentration data were fitted to the Michaelis-Menten equation using the Origin 2017 software, version b9.4.0.220 (Origin Lab, Northampton, MA, USA).

### Determination of the mutational effect on the stability of the ES^‡^ complex

The effects of the mutations on the stability of the ES^‡^ complex were determined using the equation ΔΔ*G*^‡^ = *RT*ln(*k*_cat_/*K*_m1_ / *k*_cat_/*K*_m2_), where *k*_cat_/*K*_m_ were determined for the hydrolysis of the same substrate by the mutant (1) or wild-type (2) enzyme [28; 29]. *T* is 303 K. The hydrolytic reaction catalyzed by β-glucosidases is divided in two steps, glycosylation and deglycosylation, each of them has an ES^‡^ complex, as represented in the S1 Fig [19; 29]. In that scheme *k*_cat_/*K*_m_ = (*k*_1_*k*_2_) / (*k*_2_ + *k*_-1_), corresponding to the rate constant for the glycosylation step (S1 Fig). Free energy changes (Δ*G*^‡^) calculated with this parameter relate to differences between glycosylation ES^‡^ and free enzyme and substrate. Assuming that wild-type and mutant enzymes present similar ground states, ΔΔ*G*^‡^ indicates differences between two ES^‡^ [29]. A ΔΔ*G*^‡^ higher than 3 kJ/mol was considered to be significant because this is the lower limit for destabilization of the ES^‡^ complex due to the disruption of one hydrogen bond [30].

## Results and discussion

**Fig 1:**
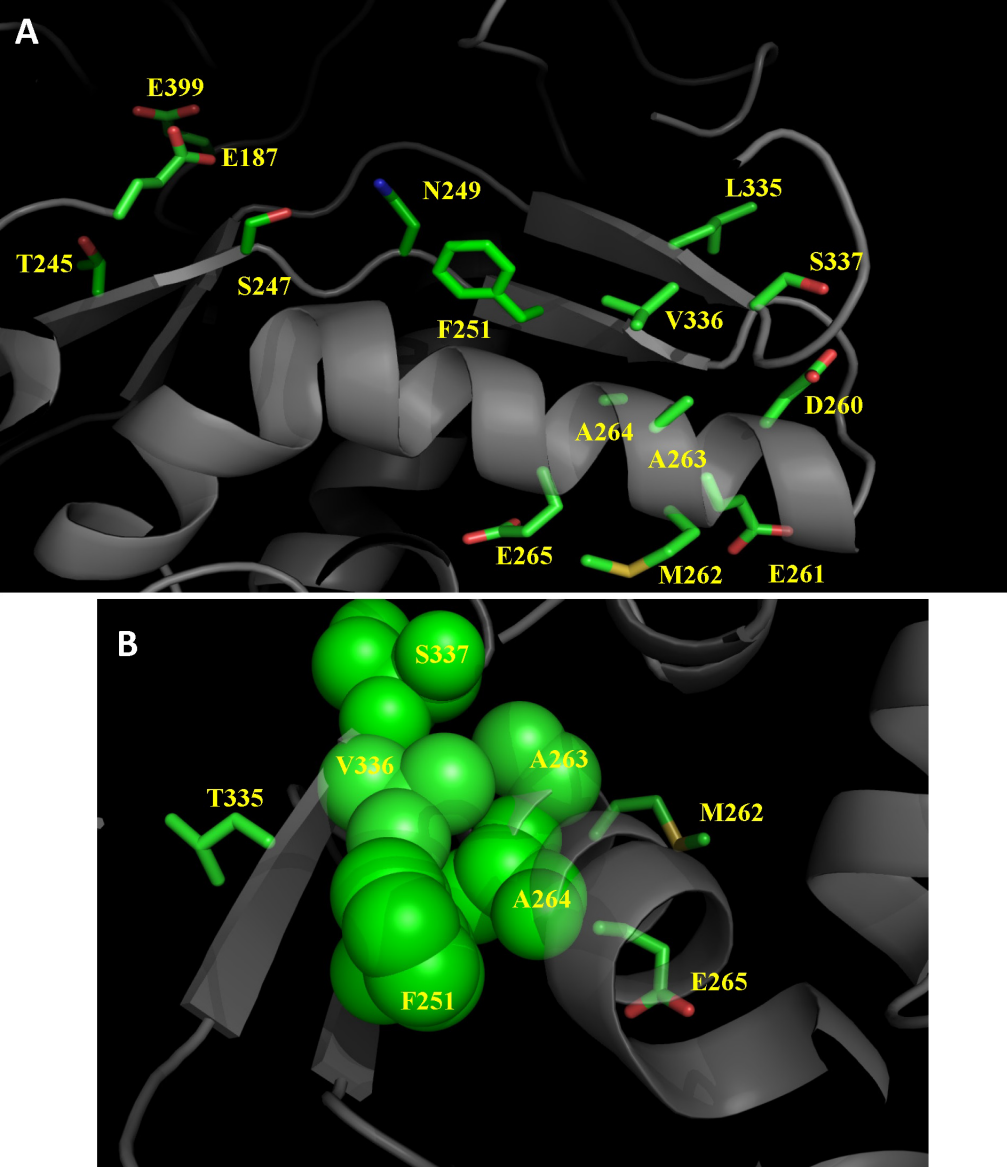
Relative structural positioning of the hub residues, mutated residues and the active site of Sfβgly (5CG0; [17]). Residues D260 to E265 and L335 to S337, which are close to the hub residue F251, were submitted to site-directed mutagenesis. F251, N249, S247 and T245 are hub residues. E187 and E399 are the catalytic acid and nucleophile, respectively. For clarity, only the side chains are shown, some secondary elements were removed and panels A and B are shown in different perspectives. **A**–The hub residues F251, N249, S247 and T245 form a pathway connecting the mutation sites near the protein surface to the core and active site of Sfβgly. The shortest distances are: F251_Cε_ –N249_Cβ_: 4.1 Å; N249Nδ2 –S247_Oλ_: 3.9 Å; S247_Cβ_-T245_Cβ_: 7.7 Å; F251_Cβ_ –A264_Cβ_: 5.3 Å; A264_Cβ_ - A263_Cβ_: 5.3 Å. These contacts, except those involving T245, are within the distance limits for noncovalent interactions (*i*.*e*., 5 Å). **B**–A different perspective of the mutated residues A263, A264, V336 and S337, which are close to the hub residue F251. The side chains are shown as spheres highlighting the clustering of A263, A264, V336, S337 and F251. The contact distances are near to the upper limit (5 Å) for non-covalent interactions (F251_Cβ_ –V336_Cλ_: 4 Å; F251_Cβ_ –A264_Cβ_: 5.3 Å; A264_Cβ_ –A263_Cβ_: 5.3 Å). Residues M262, E265 and T335, sites of innocuous mutations, do not take part of this cluster.

The PSN hub residues may effectively spread mutational perturbations through the protein structure. For instance, in the specific case of the β-glycosidase Sfβgly, mutations that perturb the environment near the PSN hub F251 reduced its thermostability [25]. To evaluate the hypothesis that PSN hub residues could influence the functional properties of enzymes, here we investigate whether the mutational perturbation spreading from the hub F251 is propagated into the Sfβgly active site, altering its catalytic properties. F251 is part of a group of hub residues (T245, S247, N249 and F251) that extends from the surface to the Sfβgly core [25] and places F251 at a short contact distance to the whole Sfβgly structure (Fig 1A). Indeed, the closeness centrality of F251 indicates that at most five contacts are necessary to link it to any residue in the Sfβgly PSN.

In the region closely surrounding F251, we found residues D260, E261, M262, A263, A264 and E265, which are part of an α-helix, and residues L335, V336 and S337, forming a β-strand (Fig 1A). The mutations analyzed in this study, D260A, E261A, M262F, A263F, A264F, E265A, L335A, V336F and S337F, introduced voids (replacements by A) or extra volume (replacements by F) in the vicinity of the central residue F251, potentially perturbing its spatial position and interactions.

Two mutants, L389A and N391A, were also studied as controls. Residues L389 and N391 are also near to a phenylanaline residue, F280. Moreover, none of these three residues (F280, L389 and N391) are hubs.

Wild-type and mutant Sfβgly were produced in the bacteria NovaBlue (DE3) and purified as previously described [25]. Circular dichroism and tryptophan fluorescence spectra confirmed their correct folding [25]. Steady state kinetic parameters for the hydrolysis of four different substrates (NPβglc, NPβfuc, NPβgal and cellobiose) were determined, except for A264F and V336F (Table 1; S2 Fig). These two mutants showed very low enzyme activity and thermal stability [25], preventing further analysis.

**Table 1:** Kinetic parameters for the substrate hydrolysis by the wild-type and mutant Sfβgly.

		kinetic parameter		
enzyme	substrate	*K*_m_ (mM)	*k*_cat_ (s^-1^)	*k*_cat_/*K*_m_
wild-type	*p*NPβGlu	0.87 ± 0.04	2.56 ± 0.02	2.9 ± 0.1
	*p*NPβFuc	0.81 ± 0.07	5.8 ± 0.2	7.1 ± 0.7
	*p*NPβGal	4.0 ± 0.2	0.322 ± 0.006	0.080 ± 0.004
	cellobiose	5.0 ± 0.6	2.14 ± 0.08	0.42 ± 0.05
D260A	*p*NPβGlu	1.05 ± 0.06	1.63 ± 0.03	1.5 ± 0.1
	*p*NPβFuc	0.82 ± 0.05	2.95 ± 0.09	3.6 ± 0.2
	*p*NPβGal	4.1 ± 0.3	0.223 ± 0.004	0.054 ± 0.004
	cellobiose	7 ± 1	1.45 ± 0.07	0.21 ± 0.03
E261A	*p*NPβGlu	0.94 ± 0.01	2.10 ±0.06	2.23 ± 0.07
	*p*NPβFuc	0.80 ± 0.06	5.3 ± 0.2	6.6 ± 0.5
	*p*NPβGal	3.6 ± 0.2	0.310 ± 0.006	0.086 ± 0.005
	cellobiose	4.5 ± 0.3	1.89 ± 0.04	0.42 ± 0.03
M262A	*p*NPβGlu	1.04 ± 0.07	2.64 ± 0.05	2.5 ± 0.2
	*p*NPβFuc	1.4 ± 0.1	7.9 ± 0.5	5.7 ± 0.5
	*p*NPβGal	3.2 ± 0.4	0.32 ± 0.01	0.10 ± 0.01
	cellobiose	4.5 ± 0.3	2.13 ± 0.04	0.47 ± 0.03
A263F	*p*NPβGlu	1.60 ± 0.06	0.806 ± 0.008	0.50 ± 0.05
	*p*NPβFuc	0.64 ± 0.06	1.35 ± 0.05	2.1 ± 0.2
	*p*NPβGal	5.2 ± 0.4	0.194 ± 0.007	0.037 ± 0.003
	cellobiose	6.8 ± 0.7	0.72 ± 0.04	0.11 ± 0.01
E265A	*p*NPβGlu	1.4 ± 0.1	1.86 ± 0.04	1.3 ± 0.1
	*p*NPβFuc	1.5 ± 0.1	6.4 ± 0.5	4.3 ± 0.4
	*p*NPβGal	4.5 ± 0.4	0.278 ± 0.008	0.062 ± 0.006
	cellobiose	7.8 ± 0.7	1.74 ± 0.07	0.22 ± 0.02
L335A	*p*NPβGlu	1.2 ± 0.1	1.57 ± 0.05	1.3 ± 0.1
	*p*NPβFuc	0.61 ± 0.05	3.4 ± 0.1	5.6 ± 0.5
	*p*NPβGal	3.7 ± 0.2	0.247 ± 0.005	0.067 ± 0.004
	cellobiose	5.8 ± 0.5	1.07 ± 0.03	0.18 ± 0.02
S337F	*p*NPβGlu	1.4 ± 0.1	0.96 ± 0.02	0.68 ± 0.05
	*p*NPβFuc	0.45 ± 0.02	1.24 ± 0.01	2.7 ± 0.1
	*p*NPβGal	5.7 ± 0.5	0.147 ± 0.005	0.026 ± 0.002
	cellobiose	7 ± 1	0.50 ± 0.03	0.07 ± 0.01
L389A	*p*NPβGlu	0.69 ± 0.06	0.76 ± 0.01	1.1 ± 0.1
	*p*NPβFuc	0.8 ± 0.1	2.1 ± 0.1	2.6 ± 0.3
	*p*NPβGal	3.2 ± 0.5	0.148 ± 0.007	0.046 ± 0.008
	cellobiose	nd	nd	nd
N391A	*p*NPβGlu	1.07 ± 0.06	1.22 ± 0.02	1.14 ± 0.07
	*p*NPβFuc	0.7 ± 0.1	2.8 ± 0.2	4.1 ± 0.6
	*p*NPβGal	4.5 ± 0.6	0.20 ± 0.01	0.044 ± 0.006
	cellobiose	nd	nd	nd

nd, not determined.

**Fig 2:**
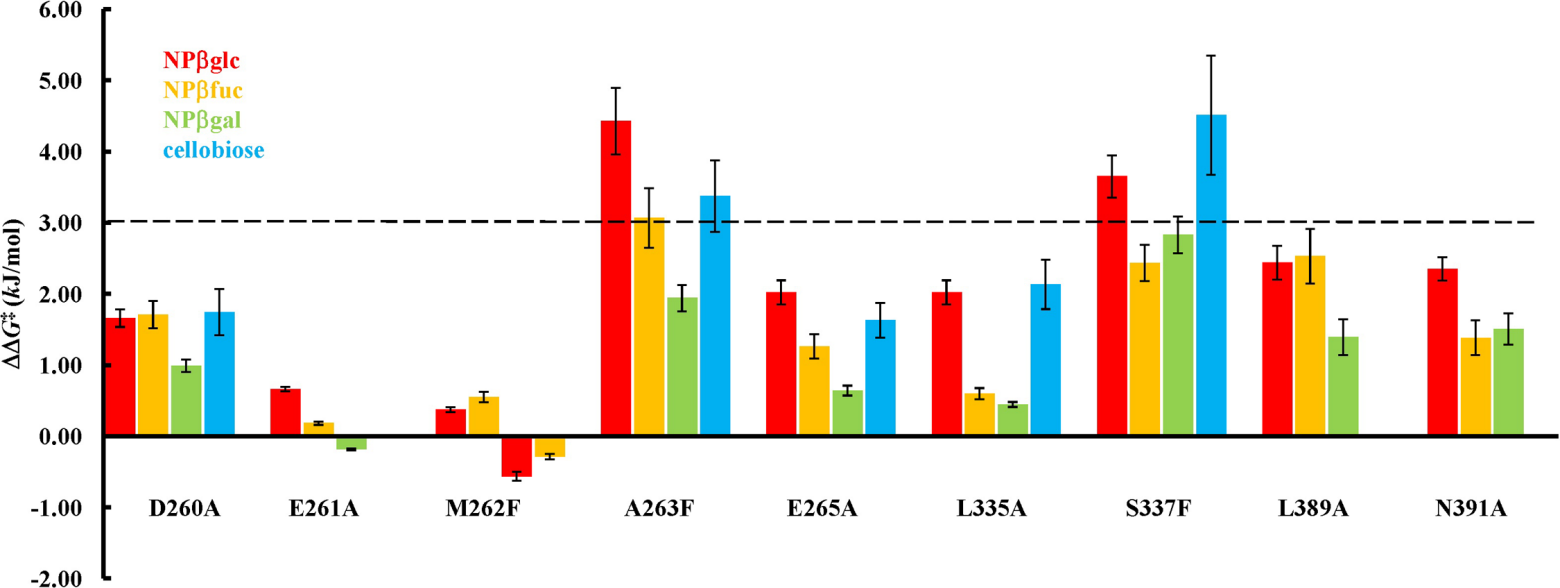
Mutational effects on the stability of the ES^‡^ complexes involving different substrates. ΔΔ*G*^‡^ were determined based on the *k*_cat_/*K*_m_ for the hydrolysis of different substrates. See the Materials and Methods for more details.

In order to determine the modifications within the Sfβgly active site that altered kinetic parameters (Table 1), the *k*_cat_/*K*_m_ ratio was used to quantify changes of activation free energy, ΔΔ*G*^‡^ [28; 29] (Table 2; Fig 2). The relative values ΔΔ*G*^‡^ based on the *k*_cat_/*K*_m_ may represent the stability difference in free energy between two ES^‡^ complexes formed by the wild-type and mutant enzyme (see also the Material and Methods). Thus, this free energy difference probes the mutational effect on interactions involving active site residues and the transition state substrate. Only a ΔΔ*G*^‡^ higher than 3 kJ/mol was considered to be significant because this is the lower limit for destabilization of the ES^‡^ complex due to the disruption of only one hydrogen bond [30].

**Table 2:** Changes in the activation free energy (ΔΔ*G*^‡^) for substrate hydrolysis due to mutations of Sfβgly.

mutation	substrate	ΔΔ*G*^*‡*^ (*k*J.mol^-1^)
D260A	*p*NPβGlu	1.7 ± 0.1
	*p*NPβFuc	1.7 ± 0.2
	*p*NPβGal	0.99 ± 0.08
	cellobiose	1.7 ± 0.3
E261A	*p*NPβGlu	0.66 ± 0.03
	*p*NPβFuc	0.18 ± 0.02
	*p*NPβGal	- 0.18 ± 0.01
	cellobiose	0
M262A	*p*NPβGlu	0.37 ± 0.03
	*p*NPβFuc	-0.55 ± 0.07
	*p*NPβGal	- 0.56 ± 0.06
	cellobiose	– 0.28 ± 0.03
A263F	*p*NPβGlu	4.4 ±0.4
	*p*NPβFuc	3.1 ± 0.4
	*p*NPβGal	1.9 ± 0.2
	cellobiose	3.4 ± 0.5
E265A	*p*NPβGlu	2.0 ± 0.2
	*p*NPβFuc	1.2 ± 0.1
	*p*NPβGal	0.64 ± 0.07
	cellobiose	1.6 ± 0.2
L335A	*p*NPβGlu	2.0 ± 0.2
	*p*NPβFuc	0.60 ± 0.07
	*p*NPβGal	0.45 ± 0.03
	cellobiose	2.1 ± 0.3
S337F	*p*NPβGlu	3.6 ± 0.3
	*p*NPβFuc	2.4 ± 0.2
	*p*NPβGal	2.8 ± 0.2
	cellobiose	4.5 ± 0.8
L389A	*p*NPβGlu	2.4 ± 0.2
	*p*NPβFuc	2.5 ± 0.4
	*p*NPβGal	1.4 ± 0.2
	cellobiose	nd
N391A	*p*NPβGlu	2.3 ± 0.2
	*p*NPβFuc	1.4 ± 0.2
	*p*NPβGal	1.5 ± 0.2
	cellobiose	nd

Positive ΔΔ*G*^‡^ values corresponds to less stable ES^‡^ complex.

nd, not determined.

Using this criteria, only mutations A263F and S337F caused significant changes in ΔΔ*G*^‡^ for the reactions involving substrates NPβglc and cellobiose (Table 2; Fig 2). Replacement of residues D260, E261, M262, E265 and L335, which are part of the same α-helix and β-strand (Fig 1), did not substantially affected the ES^‡^ complex stability. Moreover, L389A, and N391A control mutations, directed to a “non-hub” residue, did not change the Sfβgly kinetic parameters (Table 1) or activation free energy (Fig 2). Hence, A263F and S337F mutations, distant from the active site, altered the noncovalent interactions involved in transition state stabilization. Note that A263_Cβ_ and S337_Oγ_ are 25 Å away from the catalytic nucleophile E399_Oε._

In Sfβgly, the active site residue E451 forms two hydrogen bonds with the 4 and 6 hydroxyl groups of the substrate glycone (OH4 and OH6 hereafter), whereas Q39 forms a hydrogen bond with OH4 (S3 Fig). Hence, they contribute to the catalysis through the stabilization of the ES^‡^ complex [22; 24]. Residues Q39 and E451 and their interactions with S^‡^ are highly conserved among GH1 β-glucosidases, which shows their essential role in the catalytic activity and substrate specificity of those enzymes [31; 32]. Contributions of these noncovalent interactions to transition state stabilization were previously determined based on comparison of *k*_cat_/*K*_m_ for Sfβgly wild-type and mutants lacking the side chains of residues 39 and 451, Q39A and E451A respectively [22; 23].

Following that same procedure, we estimated the free energy of noncovalent interactions involving the residues Q39 and E451 and OH4 (in the equatorial and axial positions) and OH6 of the substrate for the A263F and S337F mutants (Table 3). The A263F mutation, which is distant 25 Å from the active site, caused a significant decrease in Q39-OH4_equatorial_ and Q39-OH4_axial_ interactions (5.6 *k*J/mol and 3.1 *k*J/mol, respectively). The E451-OH4_equatorial_ and E451-OH4_axial_ interactions were also weakened by the A263F mutation (Table 3). The S337F mutation, also distant 25 Å from the active site, decreased the stability of the Q39-OH4_equatorial_ and Q39-OH4_axial_ interactions (3.3 *k*J/mol and 2.4 *k*J/mol, respectively). The same reductions were observed for the E451-OH4_equatorial_ and E451-OH4_axial_ interactions. Finally, A263F and S337F mutations did not significantly change interactions involving OH6 (Table 3). In brief, these data show that specific noncovalent interactions involved in transition state stabilization within the Sfβgly active site were changed as a consequence of remote mutations S337F and A263F.

**Table 3:** Free energy estimated for noncovalent interactions involving active site residues Q39 and E451 and glycone hydroxyls 4 and 6 in the ES^‡^ complex of the wild-type and mutant Sfβgly.

	Enzyme		
	wild-type	A263F	S337F
Interaction		Δ*G*^‡^ (*k*J.mol^-1^)	
**Q39-OH4**_**equatorial**_	21.9	16.3	18.6
**Q39-OH4**_**axial**_	14.3	11.3	11.9
**Q39-OH6**	1.3	2.4	0.9
**E451-OH4**_**equatorial**_	35.7	30.2	32.5
**E451-OH4**_**axial**_	18.9	15.8	16.5
**E451-OH6**	-6.5	-5.3	-6.8

The Δ*G*^‡^ were calculated considering the disruption of the interaction. Hence positive values corresponds to an interaction that stabilize the ES^‡^ complex, whereas a negative value indicates the opposite effect.

Mutation A263F caused a larger change in the interactions involving OH4_equatorial_ compared to OH4_axial_, whereas mutation S337F similarly affected interactions with OH4_equatorial_ and OH4_axial_. Considering that OH4_equatorial_ occurs in NPβglc, whereas OH4_axial_ is present in NPβgal and NPβfuc, the preferential effect of the A263F mutation on interactions with OH4_equatorial_ should cause a change in the substrate specificity, whereas the S337F mutation should have a less severe effect on the specificity. Actually, the *k*_cat_/*K*_m_ ratio between NPβglc and NPβgal for the A263F mutant (14 ± 2) is about half of the ratio for the wild-type Sfβgly (36 ± 2), whereas the ratio for S337F (26 ± 3) is similar to the remaining mutants (20–28) and closer to the wild-type. Hence, A263F mutation really reduced the Sfβgly preference for NPβglc *versus* NPβgal.

Therefore only a subset of the studied mutations had an effect on the catalytic properties of the Sfβgly active site. Specifically, A264F and V336F drastically reduced the Sfβgly activity, while A263F and S337F only weakened the interactions that stabilize the transition state. Remarkably, side chains of A263 and A264 project from the α-helix lateral towards F251, whereas E261, M262 and E265, sites of innocuous mutations, are in the opposite side of the α-helix (Fig 1A). D260, even projecting its side chain from the same α-helix side as A263 and A264, is far from F251 by about 10 Å. Similarly, V336 and S337,which also affected catalysis, are part of a β-strand close to F251 (Fig 1A). In fact, the side chain of V336 is pointed directly at F251. The different outcomes of mutations placed in the same structural element suggest that the relative position of mutated residues to hub F251 is important.

**Fig 3:**
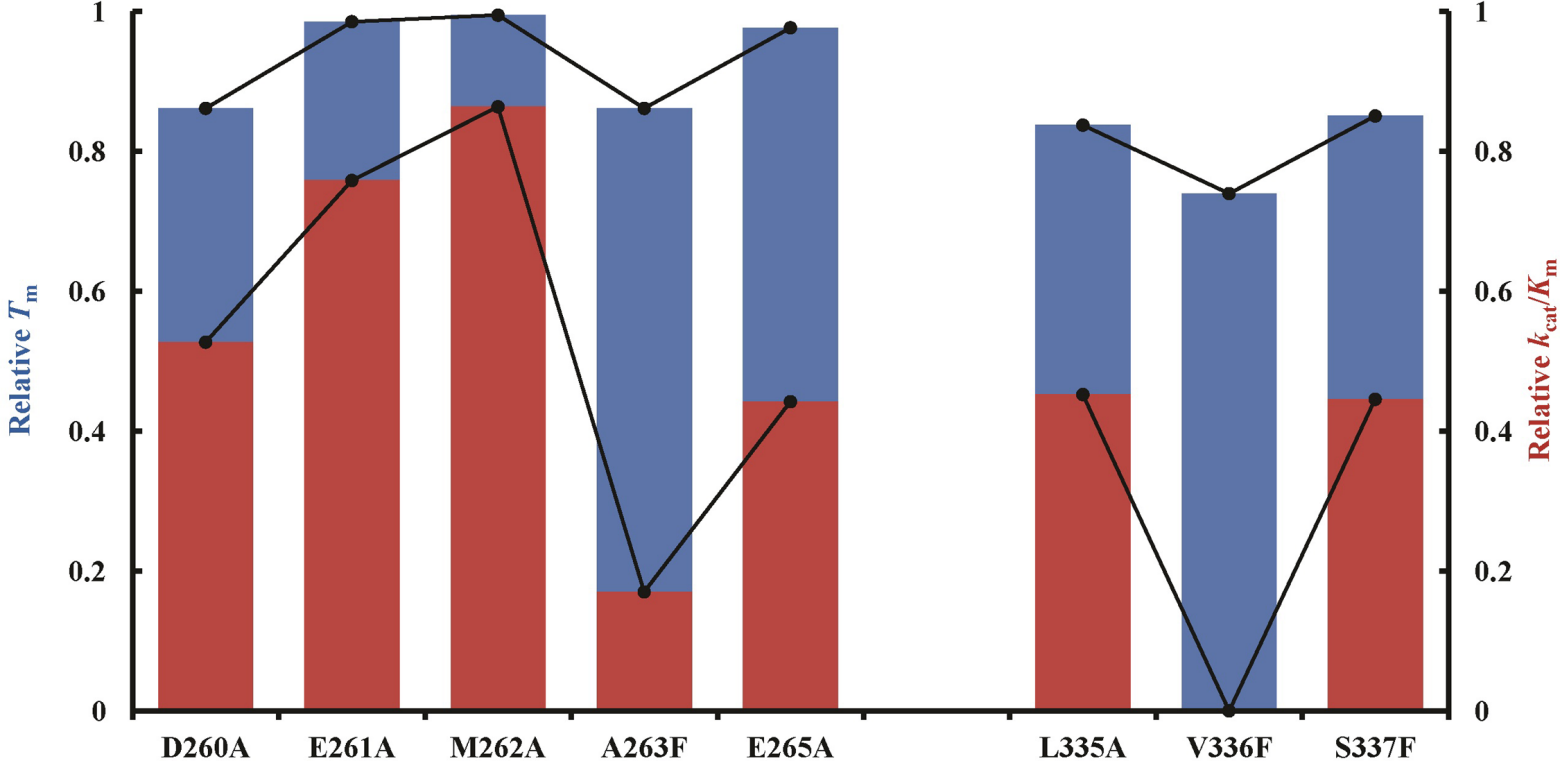
Effect of the mutations on the thermal stability (blue) and enzyme activity (red) of Sfβgly. Melting temperatures (*T*_m_) were obtained in [25]. The A264F mutant was not included because it unfolds through a multiple intermediate pathway. Hence it does not present a single *T*_m_ [25]. *k*_cat_/*K*_m_ are from Table 1. Lines were added to make it easier to follow the trends. The correlation coefficient between the Relative *T*_m_ and Relative *k*_cat_/*K*_m_ is 0.85.

A similar relationship between the spatial distribution of this same set of mutations was also observed in the analysis of the thermal stability of Sfβgly [25]. Indeed, mutational effects on the relative melting temperature (*T*_m_) and relative *k*_cat_/*K*_m_ are similar (correlation coefficient = 0.85; Fig 3), suggesting that changes in Sfβgly thermal stability and catalysis may arise from the same structural pertubations.

Residues A263, A264, V336 and S337 are closely packed, forming an internal region between the α-helix and the β-strand in which F251 is also inserted (Fig 1B). The relative solvent accessible area of these residues ranges from 0 to 10%. Therefore, replacement of the residues A263, A264, V336 and S337 by phenylalanine possibly perturbed their packing and altered the spatial positioning and conformation of residue F251.

Due to the high centrality of F251 in the Sfβgly PSN, we speculate that spatial perturbations on this residue could effectively spread and reach the active site. In detail, F251 directly contacts N249 and F334, which interacts with Y331 and W371. The side chain of W371 interacts with the substrate aglycone [17; 21], whereas Y331 contacts the catalytic nucleophile E399 and W444, which binds the glycone substrate [20; 31]. Hence, two contacts were supposedly enough to link F251 to the active site residues involved in substrate binding and catalysis. Although it is still speculative to determine details of the structural perturbations involved in the mutants, we suggest that the high centrality of F251 increased the chance that perturbations directed to it resulted in structural alterations of the active site.

## Conclusion

Mutations A263F, A264F, V336F and S337F may directly perturb the position and conformation of hub F251, which could effectively propagate these perturbations into the Sfβgly active site through short connection pathways along the protein network. This is an indication that analysis of a PSN could be valuable to predict the outcome of mutations on enzymatic activity and other protein functions.

## Supporting information

S1 Fig Kinetic scheme of the reaction catalyzed by GH1 β-glucosidases. Substrate S is formed by a monosaccharide (glycone; G) covalently linked to a group called aglycone (Ag). After the glycosidic bond cleavage, step 2, a glycosyl-enzyme intermediate (E-G) is formed and the first product, Ag, is released. In the step 3, the intermediate is hydrolyzed releasing the second product, G. Based on [19; 29].
(TIFF)

S2 Fig Effect of the concentration on the initial rate of substrate hydrolysis catalyzed by the wild-type and mutant Sfβgly. NPβglc, *p*-nitrophenyl β-glucoside; NPβgal, *p*-nitrophenyl β-galactoside; NPβfuc, *p*-nitrophenyl β-fucoside) and cellobiose. The mutants are identified in each panel. Lines represent the best fit of the data to the Michaelis-Menten equation. The substrates were prepared in 50 mM sodium citrate–sodium phosphate buffer at pH 6.
(TIFF)

S3 Fig The active site of Sfβgly. E399 is the catalytic nucleophile. Noncovalent interactions with the residues R97 and Y331 modulate ionization of E399. The residues Q39, H142 and E451 bind the substrate glycone (glc; 2-deoxy-2-fluoro-β-D-glucose). The noncovalent contact distances are: E399_Oε_ –R97_Nη_, 3.6 Å; E399_Oε_ –Y331_Oη_, 2.7 Å; E451_Oε_ –OH4, 2.2 Å; E451_Oε_ –OH6, 2.7 Å; Q39_Nε2_ –OH4, 2.7 Å; Q39_Oε1_-OH3, 2.4 Å; H142_Nε2_ –OH2, 3.0 Å. Contacts are indicated by yellow dashed lines. OH4, glycone hydroxyl 4; OH6, glycone hydroxyl 6. The active site interaction with the substrate glycone was based on the superposition of the crystallographic structures 5CG0 [17] and 1E70 [33].
(TIFF)

S1 Table Hub residues of the Sfβgly protein structure network. The central residues, also termed hub residues, of the Sfβgly PSN were identified based on the effect of their removal on the network path length (*L*). The z-score indicated the normalized increase of *L* due to the residue removal. Thus, z-scores are expressed in terms of standard deviations (σ). Cells marked in red indicate residues that are part of the Sfβgly active site. This table is based on reference [25].
(DOCX)

## Abbreviations

NPβfuc: *p*-nitrophenyl β-fucoside
NPβgal: *p*-nitrophenyl β-galactoside
NPβglc: *p*-nitrophenyl β-glucoside
PSN: Protein Structure Network
Sfβgly: β-glucosidase from *Spodoptera frugiperda*
