# Supplementary figures and images for: Mutations close to a hub residue affect the distant active site of a GH1 β-glucosidase
Source: PLoS One. 2018 Jun 6;13(6):e0198696. doi: 10.1371/journal.pone.0198696 (PMC5991390; doi:10.1371/journal.pone.0198696)

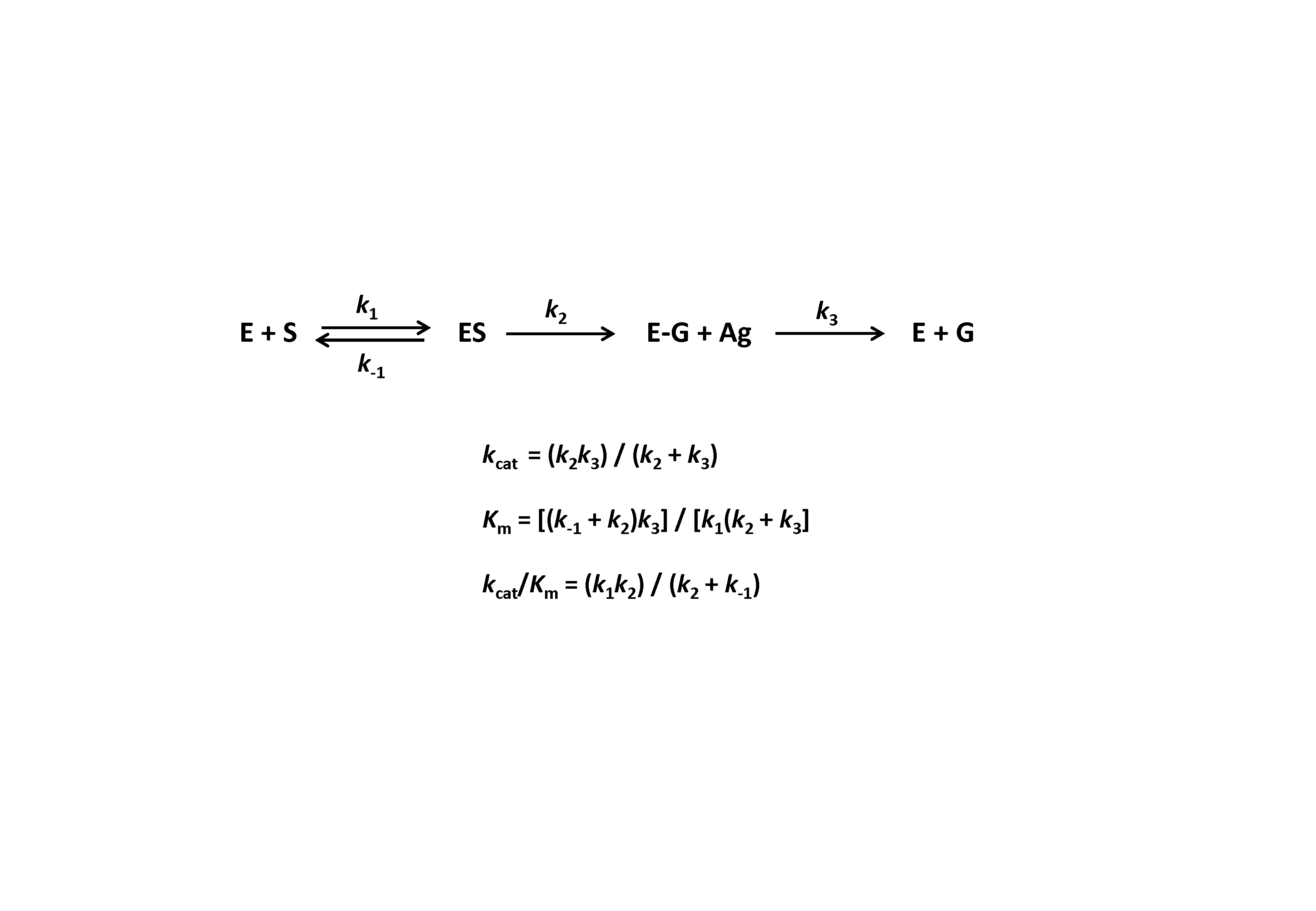

Supplement: S1 Fig — Substrate S is formed by a monosaccharide (glycone; G) covalently linked to a group called aglycone (Ag). After the glycosidic bond cleavage, step 2, a glycosyl-enzyme intermediate (E-G) is formed and the first product, Ag, is released. In the step 3, the intermediate is hydrolyzed releasing the second product, G. Based on [19; 29]. (TIFF) [file pone.0198696.s001.tiff]

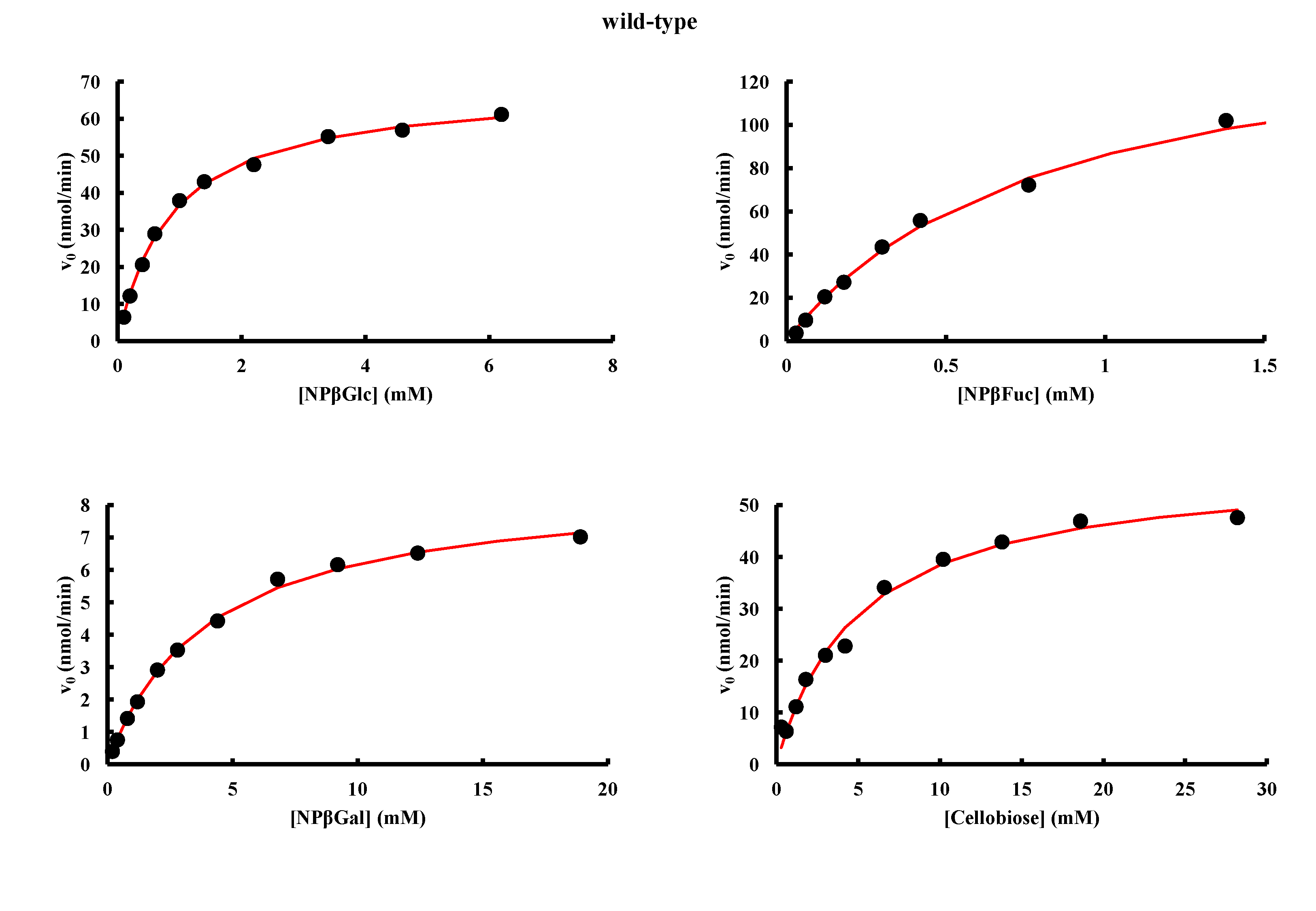

Supplement: S2 Fig — NPβglc, p-nitrophenyl β-glucoside; NPβgal, p-nitrophenyl β-galactoside; NPβfuc, p-nitrophenyl β-fucoside) and cellobiose. The mutants are identified in each panel. Lines represent the best fit of the data to the Michaelis-Menten equation. The substrates were prepared in 50 mM sodium citrate–sodium phosphate buffer at pH 6. (TIFF) [file pone.0198696.s002.tiff]

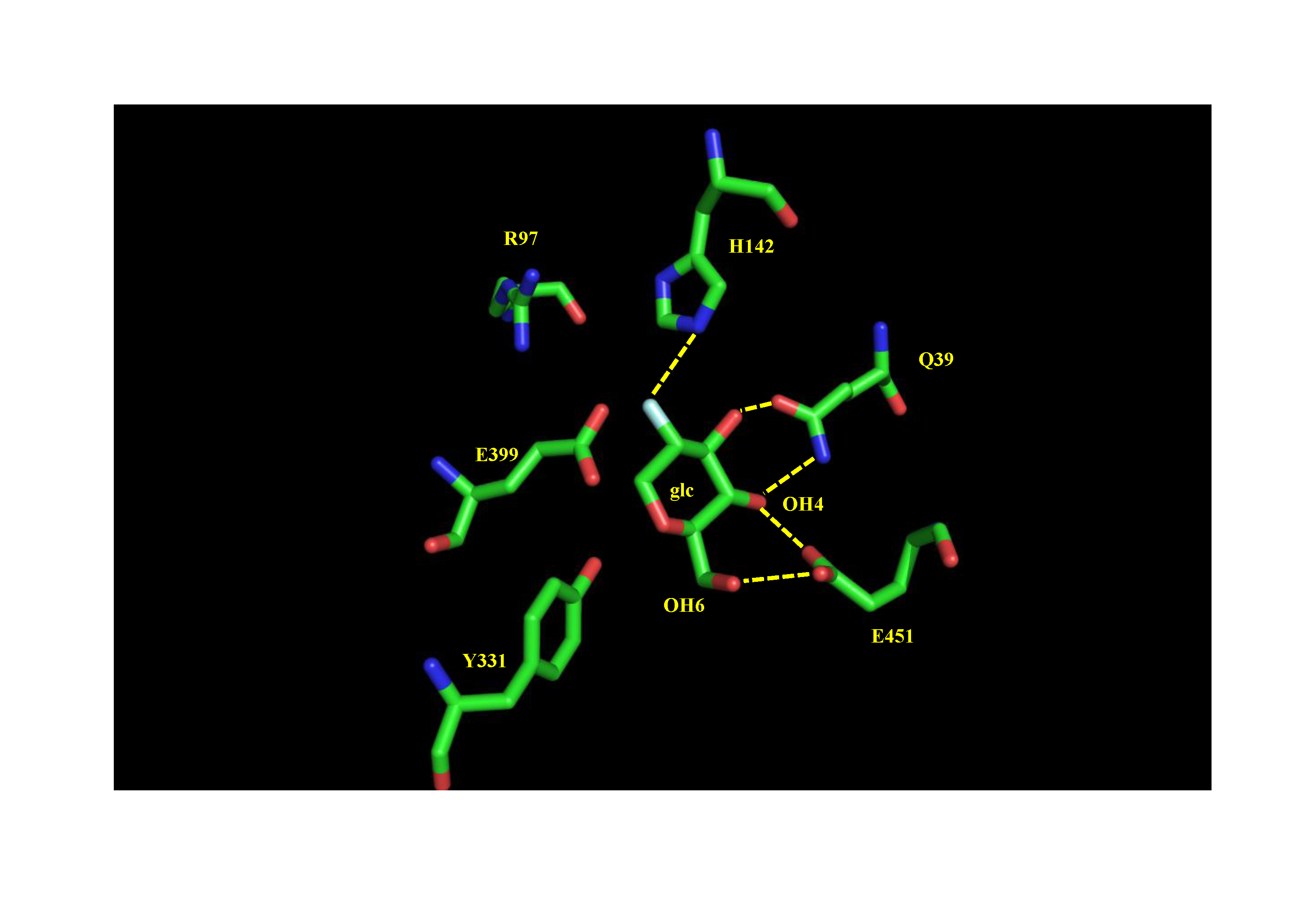

Supplement: S3 Fig — E399 is the catalytic nucleophile. Noncovalent interactions with the residues R97 and Y331 modulate ionization of E399. The residues Q39, H142 and E451 bind the substrate glycone (glc; 2-deoxy-2-fluoro-β-D-glucose). The noncovalent contact distances are: E399Oε –R97Nη, 3.6 Å; E399Oε –Y331Oη, 2.7 Å; E451Oε –OH4, 2.2 Å; E451Oε –OH6, 2.7 Å; Q39Nε2 –OH4, 2.7 Å; Q39Oε1-OH3, 2.4 Å; H142Nε2 –OH2, 3.0 Å. Contacts are indicated by yellow dashed lines. OH4, glycone hydroxyl 4; OH6, glycone hydroxyl 6. The active site interaction with the substrate glycone was based on the superposition of the crystallographic structures 5CG0 [17] and 1E70 [33]. (TIFF) [file pone.0198696.s003.tiff]
