# Supplementary material for: Mutations close to a hub residue affect the distant active site of a GH1 β-glucosidase
Source: PLoS One. 2018 Jun 6;13(6):e0198696. doi: 10.1371/journal.pone.0198696 (PMC5991390; doi:10.1371/journal.pone.0198696)
Supplement: S1 Table — The central residues, also termed hub residues, of the Sfβgly PSN were identified based on the effect of their removal on the network path length (L). The z-score indicated the normalized increase of L due to the residue removal. Thus, z-scores are expressed in terms of standard deviations (σ). Cells marked in red indicate residues that are part of the Sfβgly active site. This table is based on reference [25]. (DOCX) [file pone.0198696.s004.docx]

**Supplementary Table S1 Hub residues of the Sfβgly protein structure network**

|  | R97 | F251 | S358 | E399 | T245 | K366 | F334 | S247 | N249 | Y420 | Y331 |
| --- | --- | --- | --- | --- | --- | --- | --- | --- | --- | --- | --- |
| **z-score** | 6.4 | 6.0 | 5.1 | 4.2 | 4.2 | 4.0 | 3.6 | 3.6 | 3.5 | 3.3 | 2.9 |

The central residues, also termed hub residues, of the Sfβgly PSN were identified based on the effect of their removal on the network path length (*L*). The z-score indicated the normalized increase of *L* due to the residue removal. Thus, z-scores are expressed in terms of standard deviations (σ). Cells marked in red indicate residues that are part of the Sfβgly active site. This table is based on reference [25].
